# Near-zero effective impedance with finite phase velocity for sensing and actuation enhancement by resonator pairing

**DOI:** 10.1038/s41467-018-07697-7

**Published:** 2018-12-10

**Authors:** Kiyean Kim, Chung Il Park, Hyuk Lee, Yoon Young Kim

**Affiliations:** School of Mechanical and Aerospace Engineering, Seoul National University, 1 Gwanak-ro Gwanak-gu, 151-744 Korea

## Abstract

In spite of the extensive studies of zero-index metamaterials, the realization of zero impedance with finite phase velocity has not been explored. Here, we show that this extreme case, realized by elaborately-tuned paired resonators, can effectively enhance sensing and actuation. To explain the formation mechanism of the near-zero effective impedance with finite phase velocity by paired resonators at a target frequency, a theory using an equivalent model based on mechanical longitudinal waves is developed. If the frequency of the extreme property is further tuned at a Fabry–Pérot resonance frequency, highly efficient enhancement is possible. Experiments using a piezoceramic transducer (PZT) installed on the plate region bounded by two resonators confirm that the proposed extreme property mechanism highly enhances the sensing and actuation outputs of the transducer.

## Abstract (web summary)

In spite of extensive studies on zero-index metamaterials, the realization of zero impedance with finite phase velocity has not been explored. Here, the authors show that this extreme case, realized by elaborately-tuned paired resonators, can effectively enhance sensing and actuation.

## Introduction

The intrinsic material properties, such as density *ρ* (permeability *μ*) and stiffness *E* (permittivity *ε*), determine the phase velocity and impedance in media carrying mechanical (electromagnetic) waves. To realize extraordinary wave phenomena, these material properties have been manipulated by various means including metamaterials^1–6^. For instance, the effective impedance ($z = \sqrt {\rho E}$ or $\sqrt {\mu /\varepsilon }$) can be manipulated for perfect matching^7–10^ and the phase velocity^11–16^ ($c_{\mathrm{p}} = \sqrt {E/\rho }$ or $\sqrt {1/(\mu \varepsilon )}$) can be manipulated for tunneling and other applications. While zero-index metamaterials requiring extreme effective material properties such that *ρ*/*E* → 0 (*με* → 0) are reported^13,14,17–19^, another extreme case of zero or near-zero impedance requiring that $\sqrt {\rho E} \to 0$ $\left( {\sqrt {\mu /\varepsilon } \to 0} \right)$ is little studied. Furthermore, the realization of the near-zero impedance with finite phase velocity, which will be mainly invested here, has not been explored so far. Because the mechanical impedance *z* is the ratio of a given force *F* to the resulting particle velocity $\dot u$, the decrease in impedance for a force of fixed magnitude would increase the particle velocity (displacement). Therefore, actuation and sensing can be substantially enhanced if near-zero impedance is realized.

With extreme material properties of (*ρ* → 0 and finite *E*) or (*ρ* → 0 and *E* → ∞), zero-index metamaterials yielding the infinite phase velocity can be realized. The former case yields zero impedance (*z* → 0) while the latter case could yield finite impedance^18^ for which impedance match with a neighboring medium may be possible. Here we investigate another unexplored extreme case that *ρ* → 0 and *E* → 0, yielding *z* → 0 while *c*_p_ can be kept finite. Our analysis will show that, when an external force of a given magnitude through a transducer excites a segment of a waveguide, the radiated power output can be highly increased if the medium forming the segment has near-zero mechanical impedance and finite phase velocity (i.e., near-zero effective *z* and finite *c*_p_). If this extreme effective material state is further coupled with the Fabry–Pérot resonance, the output enhancement is most efficient. This enhancement should work for both actuation and sensing by reciprocity. A specific application of this phenomenon may be the ultrasonic excitation by a transducer for health monitoring^20–23^ in a waveguide such as the (curved) plates of an oil tank and pipelines in a nuclear power facility. For instance, a large metal plate structure is inspected with the lowest symmetric guided wave mode (*S*_0_ mode)^24^ where highly enhanced signal-to-noise ratios by this zero impedance and finite velocity phenomenon can be critically useful. In spite of a big potential application of the zero impedance concept with finite phase velocity, however, the mechanism to achieve zero or near-zero effective impedance has not been explored. Widely used metamaterials typically made of an array of periodic resonant or non-resonant unit cells do not seem to be effective in this extreme case.

Here we propose a unique mechanism to form near-zero effective impedance by using only a finite number of elaborately tuned discrete resonators. Specifically, we show that if a target segment of a medium is surrounded by paired discrete resonators, its effective impedance can become near zero while its finite phase velocity is unaltered. To verify the realization of near-zero effective impedance and show its effectiveness for highly enhanced wave actuation and sensing, we design an experiment with a mechanical longitudinal wave excited inside the region surrounded by a pair of discrete resonators. Then we show that the high enhancement is the consequence of the near-zero effective impedance observed in the wave actuated zone. Furthermore, we tune the frequency of the near-zero effective impedance at a Fabry–Pérot resonance frequency of the finite-sized effective medium for the maximized efficiency. Obviously, sensing and actuation should take place at this frequency. It is worth noting that this zero-impedance-based output enhancement method is different from a common method using impedance matching because the near-zero effective impedance makes a big contrast in impedance with a neighboring medium.

## Results

### Effects of near-zero impedance

**Fig. 1:**
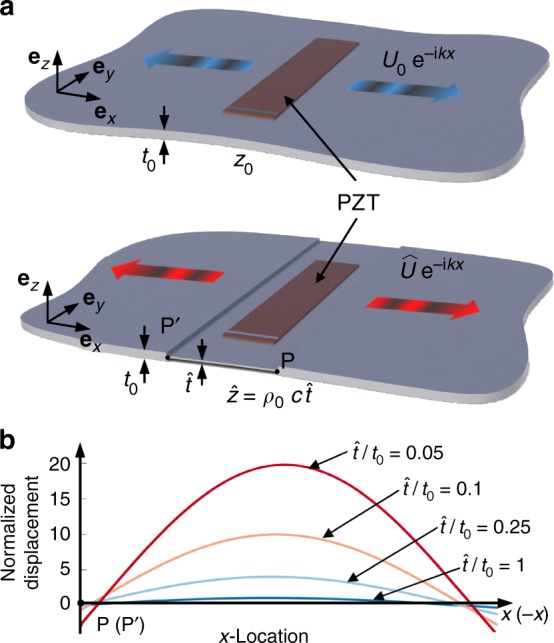
The concept of lowered impedance for wave emission enhancement. **a** Thin plates of uniform and non-uniform thicknesses carrying plane longitudinal waves. The piezoceramic patch transducers (PZT) are installed to excite the *S*_0_ Lamb waves in the plates simulating the longitudinal waves in a bar. **b** The amplification effects of the non-uniformity (expressed in terms of $\hat t/t_0$, where $\hat t$ is reduced thickness of the transducer installed zone and *t*_0_ nominal thickness) on the magnitude of the generated displacement field

To begin with a motivation to investigate the near-zero effective impedance and demonstrate its effect on wave motion, we consider the wave radiation problem depicted in Fig. 1. Figure 1a shows two thin plates functioning as waveguides carrying plane longitudinal waves propagating in the *x* direction. The radiated wave field outside of point *P* is plotted in Fig. 1b when the plates are excited by piezoceramic patch transducers. Here a Fabry–Pérot resonance frequency of the partially thinned plate is selected as the excitation frequency. Figure 1b suggests that the radiated wave field can be increased as $\hat t/t_0$ is reduced, where *t*_0_ and $\hat t$, respectively, represent the thicknesses of the nominal and machined parts of the plate. If *ρ* is understood as the line density, the reduced plate thickness corresponds to the lowered mechanical impedance. Therefore, the magnitude of power radiation $\left( {F_{{\mathrm{inp}}}^{\mathrm{2}}/2\hat z} \right)$ can increase if $\hat z$ decreases because the transducer can be regarded to provide a force of constant magnitude *F*_inp_. However, it is neither realistic nor practical to lower its impedance by machining some part of a test waveguide specimen. Hence, there ought to be a nondestructive method to lower an impedance or to even make it nearly zero.

In this work, we show that if a region of a waveguide is surrounded by a pair of resonators, its effective impedance can be lowered to nearly zero. It is well known that a single resonator can eliminate the vibrations of a harmonically excited system as a dynamic absorber^25^ and that a set of periodically arranged resonators frequently used to make metamaterials can yield an extreme density or stiffness value^26–29^. In the subsequent analysis, we will demonstrate that a pair of resonators can affect the effective impedance of the region surrounded by them. Through this investigation, it is shown that if a pair of resonators is used, the effective density and stiffness of the region that they surround vary identically as functions of the frequency. Thereby, only the effective impedance can attain an extremely low value, while the effective phase velocity remains unchanged. The frequency of near-zero effective impedance with finite phase velocity should be further tuned at a Fabry–Pérot resonance frequency; otherwise, big impedance mismatch between the region of the near-zero effective impedance medium and the surrounding origin medium prohibits wave radiation to the surrounding medium. The detailed analysis will be given below.

### Analysis of system with paired resonator

**Fig. 2:**
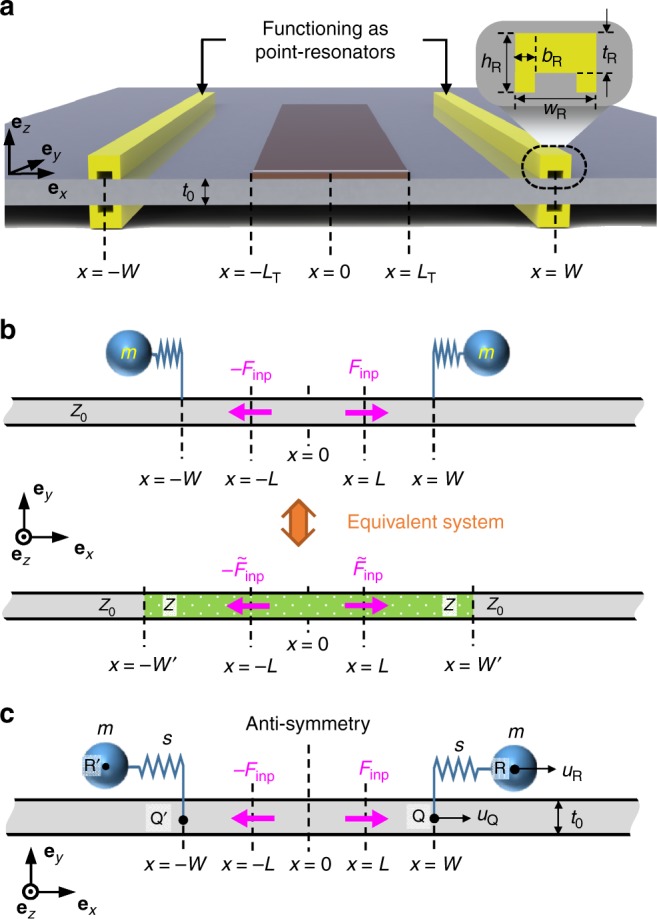
The sketch of proposed paired resonator system. **a** Sketch of a thin plate with two resonators installed at *x* = ±*W*. Each resonator at *x* = *W* or *x* = −*W* consists of two symmetrically configured C-shaped beams to ensure the generation of pure thickness-symmetric longitudinal waves without generating thickness-antisymmetric bending waves. (The dimension of the C-channel box beam aluminum resonators are *t*_R_ = 3 mm, *w*_R_ = 6 mm, *h*_R_ = 4.5 mm, *b*_R_ = 1.5 mm, and test plate thickness *t*_0_ = 2 mm.) **b** One-dimensional bar models describing the longitudinal motion in the plate shown in **a**. The model in the top illustration depicts a bar equipped with two point resonators, while the model in the bottom illustration is an equivalent bar model with a modified effective impedance to account for the effects of resonators on wave motion. The actuation by the PZT patch is modeled with two concentrated pin forces. **c** Analysis of bar model in which the displacements in various locations are specified

Figure 2a shows a thin plate with C-shaped box beams installed. The beams function as resonators. At each of the installation locations *x* = ±*W*, two beams, one on the top surface and the other on the bottom surface, are symmetrically arranged to couple with pure longitudinal waves. The waveguide is assumed to be actuated by a thin piezoceramic patch transducer with a size of 2*L*_T_ (*L*_T_ < *W*). The actual wave propagating in the plate is the lowest symmetric Lamb wave (*S*_0_) in the frequency of interest. As demonstrated in earlier works^6,16,30^, the one-dimensional longitudinal wave has a good correspondence to the *S*_0_ wave. Therefore, the wave motion in the plate will be modeled using one-dimensional longitudinal waves in a bar, as depicted in Fig. 2b. To facilitate the theoretical wave analysis, the actuation mechanism is described by a pin-force model^31,32^ using two concentrated forces (−*F*_inp_, *F*_inp_), as in Fig. 2b. This pin-force model is accurate when the mechanical impedance of the piezoceramic transducer (PZT) is negligible compared to that of the plate. To characterize accurately the actual actuation mechanism with the pin-force model, the locations of the pin forces are adjusted to (−*L*, *L*) by matching the frequency response of the PZT plate system obtained with the analytic pin-force model and that of the full finite element model. Accordingly, we used 2 *L* = 36.6 mm, while 2*L*_T_ = 30 mm.

In this analysis, each resonator can be regarded as a discrete mass-spring system coupled at a single point with the bar having the nominal mechanical impedance *z*_0_. We will show that, owing to the installation of the paired resonators, the effective impedance *z* of the region that they surround can become near-zero. To estimate the effective impedance of the surrounded region, we consider an equivalent bar model consisting of the original medium of impedance *z*_0_ and another uniform medium of lowered impedance *z*; this model is illustrated at the bottom of Fig. 2b. To construct the equivalent model, the effective length 2*W*′ defining the lowered-impedance zone should be also determined.

As depicted in Fig. 2b, two concentrated harmonic pin forces acting in opposite directions are applied at *x* = ±*L*^31,32^. They are denoted by *F*_inp_ and $\tilde F_{{\mathrm{inp}}}$ in the two models in Fig. 2b. Throughout the analysis, the harmonic dependence e^i*ωt*^ (*ω*: angular frequency, *t*: time, ${\mathrm{i}} = \sqrt { - 1}$) will be omitted. Note that $\tilde F_{{\mathrm{inp}}} \ne F_{{\mathrm{inp}}}$, where $\tilde F_{{\mathrm{inp}}}$ is the pin force in the equivalent system in which the effects of the two resonators are smeared.

To analyze the wave motion in the original one-dimensional model with two resonators, we only consider the longitudinal motion and thus use the field variables shown in Fig. 2c. The resonator consists of mass *m* and stiffness *s*, and it is attached onto the bar at point *Q* (and *Q*′). The longitudinal displacement of mass *m* is defined as *u*_R_. The displacement field in the bar will be denoted by *u*, and its value at point *Q* is denoted by *u*_Q_. Depending on the values of *x* ≥ 0, *u* is expressed using different formulas such as *u* = *u*_1_e^−i*kx*^ − *u*_1_e^i*kx*^(0 ≤ *x* ≤ *L*^−^), *u* = *u*_2_e^−i*kx*^ + *u*_3_e^i*kx*^(*L*^+^ ≤ *x* ≤ *W*^−^), and *U*e^−i*kx*^(*W*^+^ ≤ *x*). Here the frequency dependence e^i*ωt*^ is also omitted, and *k* denotes the wavenumber. Considering the field symmetry, the displacement in the bar can be written as:

1 $$u = \left\{ {\begin{array}{*{20}{l}} { - U{\mathrm{e}}^{{\mathrm{i}}kx}} \hfill & {(x \le - W)} \hfill \\ { - u_2{\mathrm{e}}^{{\mathrm{i}}kx} - u_3{\mathrm{e}}^{ - {\mathrm{i}}kx}} \hfill & {( - W \le x \le - L)} \hfill \\ {u_1{\mathrm{e}}^{ - {\mathrm{i}}kx} - u_1{\mathrm{e}}^{{\mathrm{i}}kx}} \hfill & {( - L \le x \le L)} \hfill \\ {u_2{\mathrm{e}}^{ - {\mathrm{i}}kx} + u_3{\mathrm{e}}^{{\mathrm{i}}kx}} \hfill & {(L \le x \le W)} \hfill \\ {U{\mathrm{e}}^{ - {\mathrm{i}}kx}} \hfill & {(W \le x)} \hfill \end{array}} \right.$$

The continuity and equilibrium conditions at *x* = *L* (and *x* = −*L*) yield:

2 $$u_1{\mathrm{e}}^{ - {\mathrm{i}}kL} - u_1{\mathrm{e}}^{{\mathrm{i}}kL} = u_2{\mathrm{e}}^{ - {\mathrm{i}}kL} + u_3{\mathrm{e}}^{{\mathrm{i}}kL},$$

3 $$- {\mathrm{i}}z_0\omega \,\left( {u_1{\mathrm{e}}^{ - {\mathrm{i}}kL} + u_1{\mathrm{e}}^{{\mathrm{i}}kL}} \right) = F_{{\mathrm{inp}}} - {\mathrm{i}}z_0\omega \,\left( {u_2{\mathrm{e}}^{ - {\mathrm{i}}kL} - u_3{\mathrm{e}}^{{\mathrm{i}}kL}} \right)$$

Note that the internal force considered in the force equilibrium in Eq. (3) is calculated as *EA*_0_∂*u*/∂*x*, where the stiffness *E* denotes Young’s modulus of elasticity, and *A*_0_ = *b*_0_*t*_0_ (*b*_0_: width), which is the cross-sectional area of the bar. If *ρ* is defined as the volume density, the characteristic impedance *z*_0_ is given by $A_0\sqrt {{{\rho }}E}$.

The continuity and equilibrium conditions at point *Q* yield:

4 $$u_2{\mathrm{e}}^{ - {\mathrm{i}}kW} + u_3{\mathrm{e}}^{{\mathrm{i}}kW} = U{\mathrm{e}}^{ - {\mathrm{i}}kW} \equiv u_{\mathrm{Q}},$$

5 $$- \,{\mathrm{i}}z_0\omega \left( {u_2{\mathrm{e}}^{ - {\mathrm{i}}kW} - u_3{\mathrm{e}}^{{\mathrm{i}}kW}} \right) = s(u_{\mathrm{R}} - u_{\mathrm{Q}}) - {\mathrm{i}}z_0\omega U{\mathrm{e}}^{ - {\mathrm{i}}kW}.$$

On the other hand, the equation of motion for resonator mass *m* is given by:

6 $$- m{{\omega }}^2u_{\mathrm{R}} + s(u_{\mathrm{R}} - u_{\mathrm{Q}}) = 0.$$

The expression for *U* can be obtained by solving Eqs. (2)–(6):

7 $$U = \frac{{2F_{{\mathrm{inp}}}}}{{{\mathrm{i}}z_0{{\omega }}}}\frac{{\sin kL}}{{{{\alpha }}(1 - {\mathrm{e}}^{ - 2{\mathrm{i}}kW}) - 2{\mathrm{i}}}},$$

with

8 $${{\alpha }} = \frac{1}{{z_0}}\frac{{m{{\omega }}s}}{{s{{ - }}m{{\omega }}^2}} = \frac{1}{{z_0}}\frac{{{{\omega }}s}}{{{{\omega }}_{\mathrm{R}}^2{{ - \omega }}^2}}.$$

where ${{\omega }}_{\mathrm{R}} = 2{\mathrm{\pi }}f_{\mathrm{R}} = \sqrt {s/m}$ is the resonance frequency of the resonator. If no resonator is installed (i.e., *m* = 0 or *s* = 0), the resulting displacement will be denoted as *U*_0_:

9a $$U_0 = \frac{{F_{{\mathrm{inp}}}}}{{z_0{{\omega }}}}{\mathrm{sin}}\,kL.$$

The strain that is defined as *S* = ∂*u*/∂*x* = −i*kU* (*S*_0_ = −i*kU*_0_) is more convenient to use because the PZT transducer generates and measures the strain^33^:

9b $$S = - k\frac{{2F_{{\mathrm{inp}}}}}{{z_0{{\omega }}}}\frac{{{\mathrm{sin}}kL}}{{{{\alpha }}(1 - {\mathrm{e}}^{ - 2{\mathrm{i}}kW}) - 2{\mathrm{i}}}} = - \frac{{2F_{{\mathrm{inp}}}}}{{EA_0}}\frac{{{\mathrm{sin}}kL}}{{{{\alpha }}(1 - {\mathrm{e}}^{ - 2{\mathrm{i}}kW}) - 2{\mathrm{i}}}},$$

and

9c $$S_0 = - {\mathrm{i}}\frac{{kF_{{\mathrm{inp}}}}}{{z_0{{\omega }}}}{\mathrm{sin}}\,kL = - {\mathrm{i}}\frac{{F_{{\mathrm{inp}}}}}{{EA_0}}\sin kL.$$

The strain magnitude |*S*_0_| will reach its maximum value $\left| {S_0^{f_{\mathrm{T}}}} \right| = \left| {F_{\mathrm{inp}}} \right|/EA_0$ at the frequency of *f*_T_ = *c*/4*L*, which corresponds to *kL* = π/2. Here |*F*_inp_| is assumed to be frequency independent.

### Analysis of equivalent system using effective impedance

The wave behavior observed in the original model shown at the top of Fig. 2b can also be analyzed using the equivalent model shown at the bottom of Fig. 2b. In the equivalent model, we must estimate the new effective impedance *z* of the region confined between *x* = −*W*′ and *x* = *W*′. The displacement field $\tilde u$ in the equivalent model shown in Fig. 2b may be expressed as

10 $$\tilde u = \left\{ {\begin{array}{*{20}{l}} { - \,\tilde U{\mathrm{e}}^{{\mathrm{i}}kx}} \hfill & {(x \le - W\prime )} \hfill \\ { - \,\tilde u_2{\mathrm{e}}^{{\mathrm{i}}kx} - \tilde u_3{\mathrm{e}}^{ - {\mathrm{i}}kx}} \hfill & {( - W\prime \le x \le - L)} \hfill \\ {\tilde u_1{\mathrm{e}}^{ - {\mathrm{i}}kx} - \tilde u_1{\mathrm{e}}^{{\mathrm{i}}kx}} \hfill & {( - L \le x \le L)} \hfill \\ {\tilde u_2{\mathrm{e}}^{ - {\mathrm{i}}kx} + \tilde u_3{\mathrm{e}}^{{\mathrm{i}}kx}} \hfill & {(L \le x \le W\prime )} \hfill \\ {\tilde U{\mathrm{e}}^{ - {\mathrm{i}}kx}} \hfill & {(W\prime \le x)} \hfill \end{array}} \right.$$

The field variables $\tilde u_j$ (*j* = 1, 2, 3) and $\tilde U$ are related to each other by the continuity and equilibrium conditions at *x* = *L* and *x* = *W*′ as

11 $$\tilde u_1{\mathrm{e}}^{ - {\mathrm{i}}kL} - \tilde u_1{\mathrm{e}}^{{\mathrm{i}}kL} = \tilde u_2{\mathrm{e}}^{ - {\mathrm{i}}kL} + \tilde u_3{\mathrm{e}}^{{\mathrm{i}}kL}$$

12 $$- {\mathrm{i}}z{{\omega }}\left( {\tilde u_1{\mathrm{e}}^{ - {\mathrm{i}}kL} + \tilde u_1{\mathrm{e}}^{{\mathrm{i}}kL}} \right) = \tilde F_{\mathrm{inp}} - {\mathrm{i}}z{{\omega }}\left( {\tilde u_2{\mathrm{e}}^{ - {\mathrm{i}}kL} - \tilde u_3{\mathrm{e}}^{{\mathrm{i}}kL}} \right)$$

13 $$\tilde u_2{\mathrm{e}}^{ - {\mathrm{i}}kW\prime } + \tilde u_3{\mathrm{e}}^{{\mathrm{i}}kW\prime } = \tilde U{\mathrm{e}}^{ - {\mathrm{i}}kW\prime }$$

14 $$- {\mathrm{i}}z{{\omega }}\left( {\tilde u_2{\mathrm{e}}^{ - {\mathrm{i}}kW\prime } - \tilde u_3{\mathrm{e}}^{{\mathrm{i}}kW\prime }} \right) = - {\mathrm{i}}z_0{{\omega }}\tilde U{\mathrm{e}}^{ - {\mathrm{i}}kW\prime }$$

Because the size *L* of the PZT patch should remain the same in the equivalent and the original systems, the wavenumber *k* for the region of −*L* ≤ *z* ≤ *L* should also be the same both in the equivalent and original systems. As the equivalent system is regarded as a homogeneous effective medium, the same *k* should be used over the entire equivalent system, as in Eqs. (10)–(14).

Our approach to evaluate *z* and *W'* is to make the wave field in the equivalent system equal to that in the original system with two point resonators. Accordingly, we require that the following conditions be fulfilled:

15 $$\tilde U = U,({\mathrm{for}}\,x \ge W\prime ),$$

16 $$\frac{{\tilde u_1}}{{u_1}} = \frac{{\tilde u_2}}{{u_2}} = \frac{{\tilde u_3}}{{u_3}} = g({{\omega }}),$$

17 $$\frac{{\tilde F_{{\mathrm{inp}}}}}{{F_{{\mathrm{inp}}}}} = h({{\omega }}),$$

where *g*(*ω*) and *h*(*ω*) are unknown functions of *ω* to be determined for the exact equivalence. Based on the analysis given in Supplementary Note 1, it can be shown that waves in the equivalent system behave in the same way as those in the original system if the following relations hold:

18a, b $$\frac{z}{{z_0}} = \frac{{1 - \left| r \right|}}{{1 + \left| r \right|}}\,{\mathrm{and}}\,W\prime = W + \frac{1}{{2k}}\left[ { - \beta + (2{{n}} + 1){\mathrm{\pi }}} \right],\left( {n:{\mathrm{integer}}} \right)$$

19a, b $$\frac{z}{{z_0}} = \frac{{1 + \left| r \right|}}{{1 - \left| r \right|}}\,{\mathrm{and}}\,W\prime = W + \frac{1}{{2k}}\left( { - \beta + 2{{n}}{\mathrm{\pi }}} \right),\left( {n:{\mathrm{integer}}} \right)$$

20a, b $$g(\omega ) = \sqrt {\frac{{z_0}}{z}} {\mathrm{e}}^{{\mathrm{i}}(\beta + p{\mathrm{\pi }}/2)},h(\omega ) = \sqrt {\frac{z}{{z_0}}} {\mathrm{e}}^{{\mathrm{i}}(\beta + p{\mathrm{\pi }}/2)},(p = {\mathrm{sign}}(\omega - \omega _{\mathrm{R}}))$$

where *r* and *β* are defined as

21a $$r = \frac{{{{\omega }}s}}{{2{\mathrm{i}}z_0({{\omega }}_{\mathrm{R}}^2 - {{\omega }}^2) - {{\omega }}s}},$$

21b $$\beta = \arg (r).$$

Equations (18)–(19) show that *z* and *W*′ vary as functions of *ω*, *m*, and *s*, while Eq. (18a) yields an effective impedance *z* that is smaller than *z*_0_, Eq. (19a) yields a value of *z* that is larger than *z*_0_. Because we are interested in the case where *z* < *z*_0_ given in Eq. (18a), the effective length *W'* should be estimated from Eq. (18b). Equation (18) shows that the solution for *W*′ is not unique, but it is possible to select a value close to *W* for convenience. It should be noted that, if *W'* = *W*, the magnitude and phase of $\tilde U$ cannot be the same as those of *U*. (Moreover, it can also be shown that the solution in Eq. (19) also magnifies the radiated *U* field, but we use Eq. (18) here because our work is motivated by the realization of a near-zero or lowered effective impedance.)

Using the above analysis, it is possible to derive the explicit formula for *U* (for *x* > *W'*) as

22 $$\begin{array}{*{20}{l}} U \hfill & = \hfill & {\tilde U = \frac{{\tilde F_{{\mathrm{inp}}}{\mathrm{sin}}kL}}{{z_0{{\omega }}}}\frac{{z_0{\mathrm{e}}^{{\mathrm{i}}kW\prime }}}{{{\mathrm{i}}z_0{\mathrm{sin}}kW\prime + z\cos kW\prime }}} \hfill \\ {} \hfill & = \hfill & {U_0\frac{{\sqrt {zz_0} {\mathrm{e}}^{{\mathrm{i}}(kW\prime {{ + \beta }} + p{\boldsymbol{\pi }}/2)}}}{{{\mathrm{i}}z_0{\mathrm{sin}}kW\prime + z{\mathrm{cos}}kW\prime }},\left( {p = {\mathrm{sign}}\left( {w - w_{\mathrm{R}}} \right)} \right)} \hfill \end{array}.$$

where *U*_0_ is the nominal displacement defined in Eq. (9). From Eq. (22), the following can be derived (see Supplementary Note 1):

23 $$S = \tilde S = S_0\frac{{\sqrt {zz_0} {\mathrm{e}}^{{\mathrm{i}}(kW\prime {{ + \beta }} + p{\boldsymbol{\pi }}/2)}}}{{{\mathrm{i}}z_0{\mathrm{sin}}kW\prime + z\cos kW\prime }},(p = {\mathrm{sign}}(w - w_{\mathrm{R}})).$$

Because the near-zero impedance can increase the radiated field, as demonstrated in Fig. 1, we also aim to increase |*S*| in Eq. (23). Therefore, it is possible to consider the case where both |*S*_0_| and $\sqrt {zz_0} /\sqrt {z_0^2{\mathrm{sin}}^2kW\prime + z^2{\mathrm{cos}}^2kW\prime }$ are maximized. (Because we can experimentally measure |*S*|, an analysis of |*S*| is needed to estimate *z*.) If *f* = *f*_T_ is selected, |*S*_0_| will be the largest, which is denoted by $\left| {S_0^{f_{\mathrm{T}}}} \right|$. Therefore, the expression for the normalized magnitude $\left| {S/S_0^{f_{\mathrm{T}}}} \right|$ becomes

24 $$\left| {\frac{{S({{\omega }})}}{{S_0^{f_{\mathrm{T}}}}}} \right| = \frac{{\sqrt {zz_0} }}{{\sqrt {z_0^2{\mathrm{sin}}^2kW\prime + z^2{\mathrm{cos}}^2kW\prime } }}\left| {\frac{{S_0({{\omega }})}}{{S_0^{f_{\mathrm{T}}}}}} \right|.$$

Equation (24) reveals that |*S*| can be amplified by a factor of $\sqrt {z_0/z}$ compared to nominal amplitude |*S*_0_| as long as ${\mathrm{sin}}kW\prime = 0$ is satisfied. Because *k* = *ω*/*c* (*c*: phase velocity) and *W'* = *W'*(*ω*, *m*, *s*, *W*), $\left| {S/S_0^{f_{\mathrm{T}}}} \right|$ can always be amplified at some frequencies. Furthermore, the frequency satisfying ${\mathrm{sin}}kW\prime = 0$ can be adjusted to match *f*_T_. This means that it is always possible to select a value of *W* that maximizes $\left| {S/S_0^{f_{\mathrm{T}}}} \right|$ at *f* = *f*_T_, yielding the maximally amplified radiated wave field. The maximally enhanced radiated wave field is possible because *z* becomes smaller than *z*_0_.

At this point, it is worth explaining how the effective impedance in the region bounded by the resonator pair near their resonance frequency approaches zero. To this end, an analogy will be made between the wave reflection inside the original region bounded by the resonator pair and the wave reflection inside the region of a lowered effective medium in the equivalent system. First we note that the resonators near their resonance frequency work as dynamic absorbers, making the displacement of the plate nearly zero at the point of the resonator installation. Therefore the wave *u*_2_ propagating towards the resonator is mostly reflected at the point, resulting in *u*_3_e^i*kW*^ ≈ −*u*_2_e^−i*kW*^. The wave is reflected with out of phase as if the region bounded by the resonators was surrounded by a medium of much higher impedance compared with the impedance of the bounded region. Since *ω* ≈ *ω*_R_, *r* ≈ −1 and *β* ≈ π by Eqs (21a, b). Then e^±i*kW*^ ≈ e^±i*kW*′^ by Eq. (18b) and $\tilde u_3{\mathrm{e}}^{{\mathrm{i}}kW\prime } \approx - \tilde u_2{\mathrm{e}}^{ - {\mathrm{i}}kW\prime }$ by Eq. (16). Here $\tilde u_2$ can be viewed as an incident from the medium of impedance *z* towards the medium of impedance *z*_0_ in the equivalent system while $\tilde u_3$, a reflected wave. Because the condition of $\tilde u_3{\mathrm{e}}^{{\mathrm{i}}kW\prime } \approx - \tilde u_2{\mathrm{e}}^{ - {\mathrm{i}}kW\prime }$ is satisfied at a hard wall boundary, the impedance *z* can be regarded to reach a near-zero value because the impedance value of *z*_0_ is finite.

### Effects of finite phase velocity on output power

At this point, we will explain why the condition of finite phase velocity is critical for the enhanced output power from a transducer in a medium of near-zero effective impedance. Using the expression in Eq. (9a) for the output displacement *U*_0_ by a transducer in a medium of impedance *z*_0_ without any resonator installed, one can write the output displacement *U* in a medium of impedance *z* as:

25 $$\left| U \right| = \left| {\frac{{F_{{\mathrm{inp}}}}}{{z{{\omega }}}}\sin kL} \right| = \frac{{F_{{\mathrm{inp}}}}}{{z{{\omega }}}}\left| {\sin \frac{{\omega L}}{c}} \right|.$$

The following two cases will be now considered:

#### Case 1:

zero impedance $\left( {z = \sqrt {\rho E} A_0 \to 0} \right)$ and infinite phase velocity $\left( {c = \sqrt {E/\rho } \to \infty } \right)$.

#### Case 2

(proposed case): zero impedance $\left( {z = \sqrt {\rho E} A_0 \to 0} \right)$ and finite phase velocity $\left( {c = \sqrt {E/\rho } = {\mathrm{finite}}} \right)$.

In terms of *ρ* (density) and *E* (stiffness), Cases 1 and 2 correspond to finite *E* and zero *E*, respectively, while zero *ρ* applies to both cases. Note that, for the subsequent analysis, *F*_inp_ is assumed be a fixed finite value.

First, we examine the output displacement |*U*| and power *P* as *ρ* → 0 with finite *E*. From Eq. (25),

26 $$\begin{array}{*{20}{c}} {} \\ {\lim } \\ {\rho \to 0} \\ {E = {\mathrm{finite}}} \end{array}\left| U \right| = \begin{array}{*{20}{c}} {} \\ {\lim } \\ {\rho \to 0} \\ {E = {\mathrm{finite}}} \end{array}\frac{{F_{\mathrm{inp}}}}{{z{{\omega }}}}\left| {\sin \frac{{\omega L}}{c}} \right| \approx \begin{array}{*{20}{c}} {} \\ {\lim } \\ {\rho \to 0} \\ {E = {\mathrm{finite}}} \end{array}\frac{{F_{\mathrm{inp}}L}}{{zc}},$$

where $\sin \omega L/c \approx \omega L/c$ is used because *c* → ∞ as *ρ* → 0 with finite *E*. Eq. (26) can be further simplified by using $z = \sqrt {\rho E} A_0$ and $c = \sqrt {E/\rho }$:

27 $$\begin{array}{*{20}{c}} {} \\ {\lim } \\ {\rho \to 0} \\ {E = {\mathrm{finite}}} \end{array}\left| U \right| \approx \begin{array}{*{20}{c}} {} \\ {\lim } \\ {\rho \to 0} \\ {E = {\mathrm{finite}}} \end{array}\frac{{F_{\mathrm{inp}}L}}{{zc}} \\ = \begin{array}{*{20}{c}} {} \\ {\lim } \\ {\rho \to 0} \\ {E = {\mathrm{finite}}} \end{array}\frac{{F_{\mathrm{inp}}L}}{{\sqrt {\rho E} A_0\sqrt {E/\rho } }} = \frac{{F_{\mathrm{inp}}L}}{{EA_0}}.$$

Equation (27) suggests that |*U*| remains to be finite as *ρ* → 0 and *E* = finite. Considering the output power *P* in the limit,

28 $$\begin{array}{*{20}{c}} {} \\ {\lim } \\ {\rho \to 0} \\ {E = {\mathrm{finite}}} \end{array}P = \begin{array}{*{20}{c}} {} \\ {\lim } \\ {\rho \to 0} \\ {E = {\mathrm{finite}}} \end{array}\frac{1}{2}z\left| {{\mathrm{i}}\omega U} \right|^2 \\ \approx \begin{array}{*{20}{c}} {} \\ {\lim } \\ {\rho \to 0} \\ {E = {\mathrm{finite}}} \end{array}\frac{1}{2}\sqrt {\rho E} A_0\left| {\frac{{\omega F_{\mathrm{inp}}L}}{{EA_0}}} \right|^2 \to 0.$$

The result in Eq. (28) is an indication that the power output vanishes in the limit of zero impedance and infinite phase velocity.

Second, we investigate |*U*| and *P* for Case 2 (zero impedance and finite phase velocity). Using Eq. (26) and assuming that *ω* and *L* are properly selected so that $\left| {\sin \omega L/c} \right| = 1$,

29 $$\begin{array}{*{20}{c}} {} \\ {\lim } \\ {z \to 0} \\ {c = {\mathrm{finite}}} \end{array}\left| U \right| = \begin{array}{*{20}{c}} {} \\ {\lim } \\ {z \to 0} \\ {c = {\mathrm{finite}}} \end{array}\frac{{F_{\mathrm{inp}}}}{{z{{\omega }}}}\left| {\sin \frac{{\omega L}}{c}} \right| = \begin{array}{*{20}{c}} {} \\ {\lim } \\ {z \to 0} \\ {c = {\mathrm{finite}}} \end{array}\frac{{F_{\mathrm{inp}}}}{{z{{\omega }}}} \to \infty$$

30 $$\begin{array}{*{20}{c}} {} \\ {\lim } \\ {z \to 0} \\ {c = {\mathrm{finite}}} \end{array}P = \begin{array}{*{20}{c}} {} \\ {\lim } \\ {z \to 0} \\ {c = {\mathrm{finite}}} \end{array}\frac{1}{2}z\left| {{\mathrm{i}}\omega U} \right|^2 = \begin{array}{*{20}{c}} {} \\ {\lim } \\ {z \to 0} \\ {c = {\mathrm{finite}}} \end{array}\frac{1}{2}z\left| {\frac{{F_{\mathrm{inp}}}}{z}} \right|^2 \to \infty$$

Comparing the expressions in Eqs. (28) and (30), the finite phase velocity is critical to enhance the power output of a transducer in near-zero impedance media.

### Frequency behavior of field variables

To examine the frequency behavior of $\left| {S/S_0^{f_{\mathrm{T}}}} \right|$, we used two C-channel box beams made of aluminum, where *t*_R_ = 3 mm, *w*_R_ = 6 mm, *b*_R_ = 1.5 mm, and *h*_R_ = 4.5 mm, as shown in Fig. 2a (Young’s modulus *E* = 69 GPa, Poisson’s ratio *ν* = 0.3, and density *ρ* = 2700 kg m^−3^). They were 2 *W* = 78.8 mm away from each other and installed on a 2-mm-thick aluminum plate. In a finite element analysis (see the Methods section for more details), the mass and stiffness of the resonator were estimated to be *m* = 103.0 g and *s* = 35.3 GN m^−1^, yielding *f*_R_ = 93.1 kHz. The nominal impedance and phase velocity in the aluminum plate for plane longitudinal wave motion were *z*_0_ = 28350 kg s^−1^ and *c* = 5250 m s^−1^, respectively. A PZT patch with a size of *L* = 18.3 mm was used, for which the peak frequency *f*_T_ = *c*/4*L* was 71.7 kHz.

**Fig. 3:**
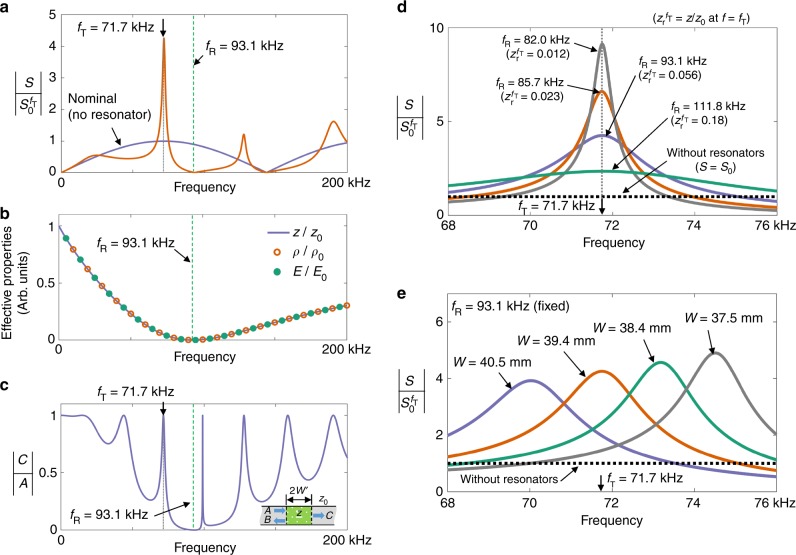
Variation of key strain fields and effective properties as function of frequency. **a** The radiated strain field |*S*|, **b** effective impedance z (density *ρ* and stiffness *E*), **c** transmission coefficient in the bar configuration shown in the inset of the bottom illustration, **d** the effects of the resonance frequency *f*_R_ on |*S*|, and **e** the effects of the distance *W* between the two resonators on |*S*|

Figure 3a, b show the behaviors of $\left| {S/S_0^{f_{\mathrm{T}}}} \right|$ and *z*/*z*_0_ as functions of the excitation frequency *f*. Note that, because *ρ* and *E* behave identically as functions of the frequency, the phase velocity remains unchanged. It is shown in Fig. 3b that *z*/*z*_0_ approaches zero as the frequency *f* approaches to *f*_R_.

Figure 3a also shows that, at *f* = *f*_T_ where the nominal output strain |*S*_0_| is maximized, |*S*| increases by a factor of 4.24 compared with the nominal value of $\left| {S_0^{f_{\mathrm{T}}}} \right|$. This amplification at *f* = *f*_T_ is due to the two facts that *z* < *z*_0_ at *f* = *f*_T_ and *f*_T_ is tuned to be one of the Fabry–Pérot resonances of the effective medium confined within the width of 2*W'*. In fact, there is a set of Fabry–Pérot resonances satisfying $\sin kW\prime = 0$. These Fabry–Pérot resonance frequencies can be more easily identified by examining the transmission coefficient |*T*| = |*C*/*A*| shown in Fig. 3c. Here *A*, *B*, and *C*, respectively, denote the magnitudes of the incident, reflected, and transmitted waves through a slab of width 2*W*′ and impedance *z* that is inserted inside a homogeneous medium of impedance *z*_0_. Equation (24) also indicates that $\left| {S/S_0} \right| = \sqrt {z_0/z} > 1$ for ${\mathrm{sin}}kW\prime = 0$ and $\left| {S/S_0} \right| = \sqrt {z/z_0} < 1$ for ${\mathrm{cos}}kW\prime = 0$. Accordingly, $\left| {S/S_0^{f_{\mathrm{T}}}} \right|$ at *f* = *f*_T_ can be amplified because *f* = *f*_T_ satisfies ${\mathrm{sin}}kW\prime = 0$.

The effects of *f*_R_ on $\left| {S/S_0^{f_{\mathrm{T}}}} \right|$ near *f* = *f*_T_ = 71.7 kHz are investigated in Fig. 3d. As *f*_R_ approaches *f*_T_, |*S*| increases rapidly because *z*_*r*_ = *z*/*z*_0_ becomes smaller (and the *Q* value becomes larger). Therefore, by tuning the value of *z* using an appropriate value for *f*_R_, a tradeoff can be always made between the amplitude and bandwidth in $\left| {S/S_0^{f_{\mathrm{T}}}} \right|$ at target frequency *f*_T_. The effect of the distance (2*W*) between the two resonators on |*S*| is shown in Fig. 3e, where *f*_R_ is assumed to be fixed. Because only *W* is varied, the effective impedance *z* does not vary. However, the Fabry–Pérot resonance frequencies in a medium of impedance *z* in the region confined between 2*W'* are varied because *W'* varies with *W*, as shown in Eq. (18). Therefore, the peak frequency of the locally maximized |*S*| is significantly affected by *W*. We argue that the phenomenon in Fig. 3e cannot be observed if only a single resonator is installed because it only functions as a dynamic absorber^25^. The wave interference occurring between the paired resonators is unique in that it can lower the effective impedance *z* of the region surrounded by the resonators, even making it nearly zero.

### Experimental results

**Fig. 4:**
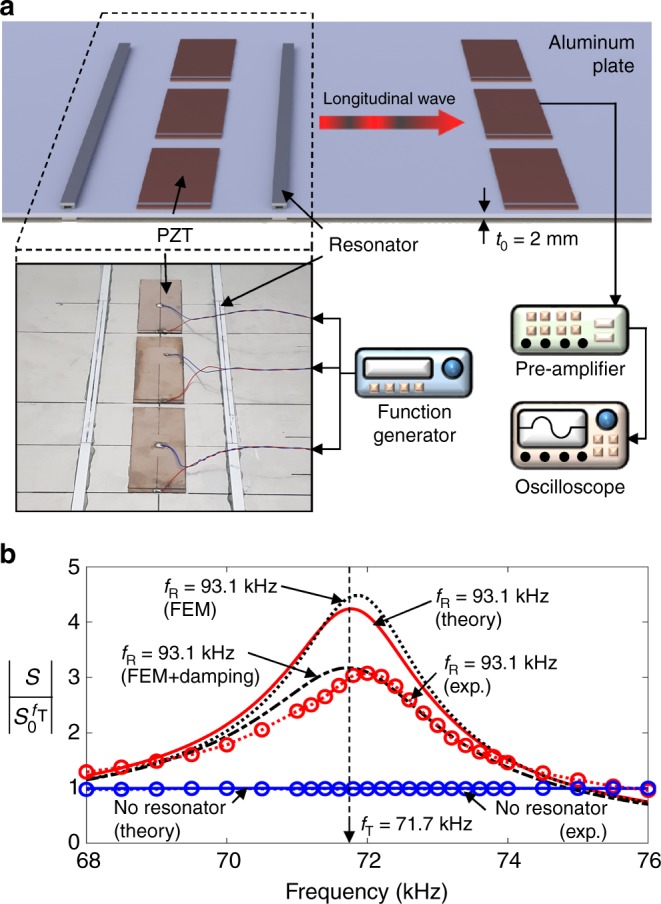
Experimental demonstration in thin plate. **a** Set-up for longitudinal wave experiments in thin plate. Three PZT patches were used to ensure the generation of a plane longitudinal wave mode in the plate. **b** A comparison of the experimental and theoretical/numerical results for the radiated strain field |*S*|

Finally, we present the results of an experiment performed to verify the near-zero impedance, or more realistically, a lowered impedance. This experiment was designed to estimate the effective impedance *z* and demonstrate the magnification of |*S*| in the radiated wave field. Figure 4a shows the experimental set-up. The geometric data and material properties used to plot Fig. 3a were also used for the experiment. The magnitude $\left| {S_0^{}} \right|$ was measured for frequencies between 68 and 76 kHz around the target frequency *f*_T_ = 71.7 kHz and it was normalized with respect to $\left| {S_0^{f_{\mathrm{T}}}} \right|$. The detailed experimental procedure is described in the Methods section. The experimental results for $\left| {S/S_0^{f_{\mathrm{T}}}} \right|$ are plotted using a red dashed line with circles in Fig. 4b. It is shown that |*S*| at *f* = *f*_T_ = 71.7 kHz is increased by 307%. The plot also shows the finite element simulation result obtained using a detailed two-dimensional continuum model, which includes the C-shaped paired resonators (having *f*_R_ = 93.1 kHz). COMSOL Multiphysics was used for the simulation. The finite element result obtained without considering any damping effect is denoted by “FEM” in Fig. 4b and is in a fairly good match to the theoretical result calculated by Eq. (9). To account for the damping effect occurring in the experiment, the loss factor of 0.065 was estimated from the experimental result and considered for the resonators in the finite element simulation. The corresponding result denoted by “FEM+damping” matches fairly well with the experimental result.

Next, we extract the value of $\left. z \right|_{\exp }$ from the experimental result at *f* = *f*_T_ using the following formula:

31 $$\frac{z}{{z_0}} = \left| {\sin ^2kL} \right|\left| {\frac{{S_0^{f_{\mathrm{T}}}}}{S}} \right|^2.$$

Equation (31) is valid when ${\mathrm{sin}}kW\prime = 0$. If the value of $\left| {S/S_0^{f_{\mathrm{T}}}} \right|$ in Fig. 4b is substituted into Eq. (31), it is possible to estimate$\left. {z/z_0} \right|_{\exp }$ = 0.053. The effect of damping is considered for the estimation. This value agrees fairly well with the theoretical value *z*/*z*_0_|_Theory_ = 0.056 at *f* = *f*_T_.

## Discussion

We found that the effective impedance *z* of a region bounded by a pair of point resonators can become near-zero, or more practically, lower than the nominal impedance *z*_0_. When the paired-resonator mechanism is used, the effective density and stiffness behave identically as functions of the frequency. Therefore, only the effective impedance can be affected, while the effective phase velocity or refractive index remains unchanged. If the frequency of a lowered effective impedance is selected to match the Fabry–Pérot resonance frequency of the equivalent system of effective impedance *z*, the wave emission by external excitation inside the region bounded by the resonators can be highly enhanced.

**Fig. 5:**
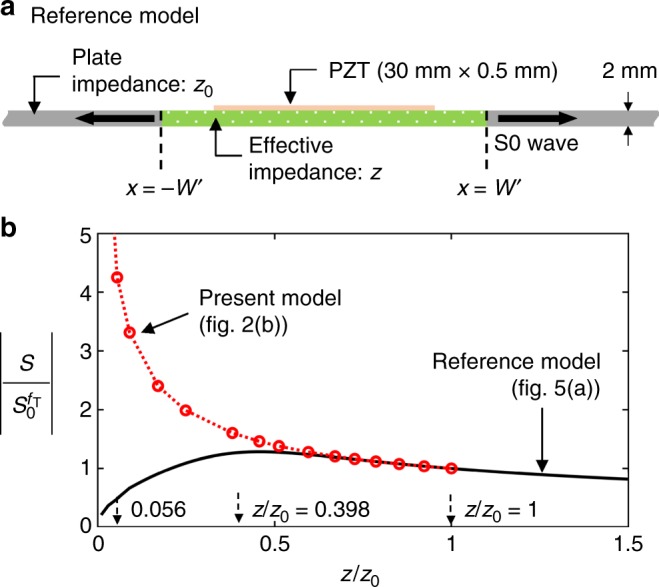
Comparison between zero-impedance concept and impedance matching concept. **a** Reference model to investigate the effects of impedance matching. It consists of a plate of nominal impedance *z*_0_ and a PZT patch of impedance *z*_PZT_ = 0.398*z*_0_ bonded onto the plate segment between *x* = −*W*′ and *x* = *W*′ having varying impedance *z*. **b** The comparison of the radiated strain field |*S*| at *f* = *f*_T_ = 71.7 kHz

To show that the high enhancement achieved in this study is not due to the impedance matching concept but due to the unique zero-impedance concept, the related simulation results are presented as Fig. 5 (See the Methods section for the detailed simulation process.). Figure 5a is a reference model considered to investigate the effects of the impedance matching where the effective impedance *z* of the plate region between *x* = −*W*′ and *x* = *W*′ is varied. This model is the counterpart of the paired-resonator model in Fig. 2b. The values of $\left| {S/S_0^{f_{\mathrm{T}}}} \right|$ for the models in Figs. 5a and 2b are compared in Fig. 5b with varying effective impedance values *z* at *f* = *f*_T_ = 71.7 kHz (the same frequency considered for Fig. 4), a Fabry–Pérot resonance frequency. Note that the impedance *z*_PZT_ of the PZT patch (*E* = 63 GPa, *ρ* = 7500 kg m^−3^) bonded on the top of the plate is *z*_PZT_/*z*_0_ = 0.398 for the model in Fig. 5a. When *z*/*z*_0_ becomes *z*_PZT_/*z*_0_ in the reference model in Fig. 5a, the strain output |*S*| becomes locally maximized, as expected from the impedance matching concept. In case when the present zero-impedance concept model is used, much larger |*S*| value than the value obtained in the impedance matched model can be obtained as |*S*| = 4.24$\left| {S_0^{f_{\mathrm{T}}}} \right|$ at *z*/*z*_0_ = 0.056.

The demonstrated high enhancement can be a critically useful application of the near-zero effective impedance because attaching paired resonators is an efficient non-destructive method to increase the transduction efficiency of any transducer for both actuation and sensing (see the Methods section for sensing). Because this method does not require the alteration of a test waveguide or an additional active element, it is not limited to the ultrasonic transducers considered as an example in this study but can open a new way to boost the efficiency of various transducers. It can also be used to block wave transmission using the realized near-zero effective impedance.

## Methods

### Realization of resonators and experimental set-up

In the experiments, we used the lowest symmetric Lamb wave (*S*_0_ wave) in a plate because it has a good correspondence to the longitudinal wave propagating in a bar, which can be modeled as a one-dimensional waveguide. Thus we assumed that the particle displacement in the plate and the motions of the resonators were all along the *x* direction. The resonators were bonded onto the plate using epoxy resin (3M DP460). The cross-sectional geometry of the C-shaped resonators in Fig. 2a was the same as that used in Fig. 4 (*t*_R_ = 3 mm, *w*_R_ = 6mm, *h*_R_ = 4.5 mm, and *b*_R_ = 1.5 mm). They were designed in this way to exhibit dominant vibrations along the *x* direction. Indeed, they functioned as mass-spring systems in the frequency range of interest.

To estimate the equivalent stiffness *s* and mass *m* of the resonator, we used its resonance frequency and static stiffness, as determined using a detailed continuum finite element model. The lowest eigenfrequency *f*_R_ was found to be 93.1 kHz in a numerical simulation. Following the procedure described in Supplementary Note 2 and Supplementary Figure 1, we estimated *s* = 35.3 GN m^−1^ from the formula *s* = *f*_*x*_/*u*_R_. Then the mass was calculated to be *m* = 103.0 g from *m* = *s*/(2π*f*_R_)^2^.

Referring to Fig. 4a, three 30 × 70 × 0.5 mm^3^ PZT patches were installed on a 2-mm-thick aluminum plate. We used three patches to ensure plane longitudinal waves. Sine pulses of 50 periods generated by a function generator (Agilent 33250A) were input to the PZT transducer for wave generation. The center frequencies were varied from 68 to 76 kHz. The frequency increments are 0.2 kHz between 71 and 74 kHz, and 0.5 kHz in elsewhere frequencies. Another set of PZT patches was installed 1.6 m away from the transmitters for sensing. The signals from the sensor were amplified through a preamplifier (SR 560) and recorded using a digital oscilloscope (LeCroy Waverunner 104MXI). The measurements were performed using a pitch-catch mode. The measured peak-to-peak voltage value *V*_0_ was proportional to the strain *S*_0_ and the sensing characteristics of the PZT patch in the plate without the resonators installed. Thus $V_0^{} = \left| {C_1} \right|\left| {S_0} \right|^2$ or $\left| {S_{{0}}} \right| = \sqrt {V_0/\left| {C_1} \right|}$, where *C*_1_ was a calibration constant. If we write $\left| {S_0^{\max }} \right| = \max (\left| {S_0} \right|)$, the normalized value can be determined to be $\left| {S_{{0}}/S_0^{\max }} \right| = \sqrt {V_0} /\max \left( {\sqrt {V_0} } \right)$. The frequency of the maximum |*S*_0_| was found to be 74 kHz in our experiment. However, around the target frequency *f* = *f*_T_ = 71.7 kHz, $\left| {S_{{0}}/S_0^{{\mathrm{max}}}} \right|$ reached a value of >0.99 or nearly the maximum value. Therefore, the discrepancy was found to be within the acceptable tolerance. If the measured peak-to-peak voltage in the plate with the installed resonators is denoted by *V*, it can also be expressed as *V* = |*C*_1_||*S*_0_||*S*|, where *S* is the corresponding strain. Therefore, it is possible to obtain a relation such that $\left| {S/S_0^{\max }} \right| = \left| {V/\left( {\sqrt {V_0} \max \left( {\sqrt {V_0} } \right)} \right)} \right|$. The results are plotted in Fig. 4b.

### Near-zero impedance for enhanced sensing

**Fig. 6:**
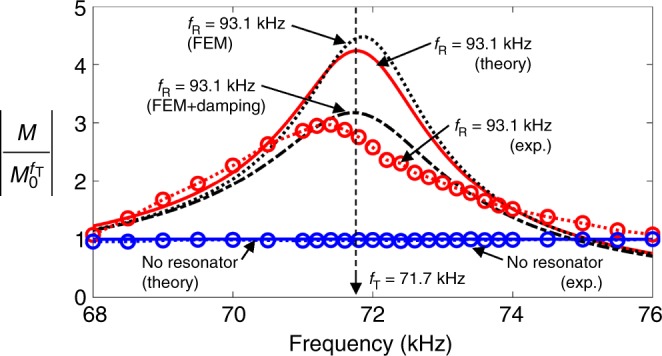
Normalized sensor output (|*M*|) relative to maximum value $\left| {M_0^{f_{\mathrm{T}}}} \right|$ in a plate without resonators. A comparison of the experimental and theoretical/numerical results for the measured sensor output |*M*|

At this time, we report the use of the near-zero effective impedance concept for sensing. For the sensing enhancement analysis and experiment, we considered exactly the same geometric and layout configurations as used for the main part of this work, including the resonance frequency *f*_R_ and target Fabry–Pérot frequency *f*_T_. In this case, the PZT transducer located inside the region bounded by the two C-shaped resonators worked as a sensing unit. Therefore, a longitudinal wave generated outside of the region was incident to the transducer. The behavior of the normalized sensor output $\left( {\left| {M/M_0^{f_{\mathrm{T}}}} \right|} \right)$ is depicted in Fig. 6. Because of the reciprocity between the actuation and sensing mechanisms, it was not surprising that the theoretical/numerical prediction of $\left| {M/M_0^{f_{\mathrm{T}}}} \right|$ for sensing was the same as the counterpart $\left| {S/S_0^{f_{\mathrm{T}}}} \right|$ for wave actuation (see Fig. 6) (Because of this reciprocity, the detailed analysis is skipped here.). It can be seen that the experimental result agrees fairly well with the numerical prediction. This analysis and experiment showed that zero effective impedance is useful for both wave actuation and sensing when a transducer is installed in a zone of lowered impedance, and the actuation/sensing frequency is properly selected.

### Simulations

First, we used the reference model shown in Fig. 5a and varied the effective impedance *z* of the plate region between *x* = −*W*′ and *x* = *W*′. Here we used *W*′ = 36.6 mm estimated from Eq. (18b) for the Fabry–Pérot resonance frequency at *f*_T_ = 71.7 kHz. The PZT patch (*E* = 63 GPa, *ρ* = 7500 kg m^−3^) sized of 30 × 0.5 mm^2^ is bonded onto the plate. Considering the actuation mechanism of a PZT patch, a uniform time-harmonic longitudinal strain was prescribed in the PZT patch. To determine the actual driving force $\tilde F_{{\mathrm{inp}}}$ in the PZT patch, we used Eq. (17) where $\left| {h(\omega )} \right| = \sqrt {z/z_0}$ by assuming that the pin force in the model in Fig. 2b is *F*_inp_. The longitudinal strain on the middle plane of the plate at any *x* > *W*′ is calculated and plotted in Fig. 5b with a black solid line.

To obtain the simulation result with the paired-resonator model in Fig. 2b, we fixed the resonant frequency *f*_R_ at 93.1 kHz and varied the values of mass *m* (simultaneously *s*) in order to consider different values of the effective impedance *z*. As in other simulations, *W* was tuned to match with the Fabry–Pérot resonance frequency at *f*_T_ = 71.7 kHz using Eq. (18b). The result of $\left| {S/S_0^{f_{\mathrm{T}}}} \right|$ is then plotted in Fig. 5b by red circles.

## Electronic supplementary material

Supplementary Information

## Data Availability

The FEM data that support the findings of this study are available in “Figshare” with the identifier 10.6084/m9.figshare.7188194.v2^34^.

## References

[1] Pendry JB (2000). Negative refraction makes a perfect lens. Phys. Rev. Lett..

[2] Shelby R, Smith D, Nemat-Nasser S, Schultz S (2001). Microwave transmission through a two-dimensional, isotropic, left-handed metamaterial. Appl. Phys. Lett..

[3] Smith DR, Schultz S, Markoš P, Soukoulis C (2002). Determination of effective permittivity and permeability of metamaterials from reflection and transmission coefficients. Phys. Rev. B.

[4] Li J, Chan C (2004). Double-negative acoustic metamaterial. Phys. Rev. E.

[5] Smith DR, Pendry JB, Wiltshire MC (2004). Metamaterials and negative refractive index. Science.

[6] Oh JH, Kwon YE, Lee HJ, Kim YY (2016). Elastic metamaterials for independent realization of negativity in density and stiffness. Sci. Rep..

[7] Kildishev AV, Narimanov EE (2007). Impedance-matched hyperlens. Opt. Lett..

[8] Xie Y, Konneker A, Popa BI, Cummer SA (2013). Tapered labyrinthine acoustic metamaterials for broadband impedance matching. Appl. Phys. Lett..

[9] Li Z (2017). Broadband gradient impedance matching using an acoustic metamaterial for ultrasonic transducers. Sci. Rep..

[10] Bok E (2018). Metasurface for water-to-air sound transmission. Phys. Rev. Lett..

[11] Bongard F, Lissek H, Mosig JR (2010). Acoustic transmission line metamaterial with negative/zero/positive refractive index. Phys. Rev. B.

[12] Choi M (2011). A terahertz metamaterial with unnaturally high refractive index. Nature.

[13] Fleury R, Alù A (2013). Extraordinary sound transmission through density-near-zero ultranarrow channels. Phys. Rev. Lett..

[14] Moitra P (2013). Realization of an all-dielectric zero-index optical metamaterial. Nat. Photonics.

[15] Zhu R, Liu X, Hu G, Sun C, Huang G (2014). Negative refraction of elastic waves at the deep-subwavelength scale in a single-phase metamaterial. Nat. Commun..

[16] Lee H, Lee JK, Seung HM, Kim YY (2017). Mass-Stiffness substructuring of an elastic metasurface for full transmission beam steering. J. Mech. Phys. Solids.

[17] Huang X, Lai Y, Hang ZH, Zheng H, Chan C (2011). Dirac cones induced by accidental degeneracy in photonic crystals and zero-refractive-index materials. Nat. Mater..

[18] Dubois M, Shi C, Zhu X, Wang Y, Zhang X (2017). Observation of acoustic Dirac-like cone and double zero refractive index. Nat. Commun..

[19] Liberal I, Engheta N (2017). Near-zero refractive index photonics. Nat. Photonics.

[20] Lowe MJ, Alleyne DN, Cawley P (1998). Defect detection in pipes using guided waves. Ultrasonics.

[21] Hirao M, Ogi H (1999). An SH-wave EMAT technique for gas pipeline inspection. NDT E Int..

[22] Chaki S, Bourse G (2009). Guided ultrasonic waves for non-destructive monitoring of the stress levels in prestressed steel strands. Ultrasonics.

[23] Wang X, Peter WT, Mechefske CK, Hua M (2010). Experimental investigation of reflection in guided wave-based inspection for the characterization of pipeline defects. NDT E Int..

[24] Wilcox P, Lowe M, Cawley P (2005). Omnidirectional guided wave inspection of large metallic plate structures using an EMAT array. IEEE T. Ultrason. Ferr..

[25] Tse, F. S., Morse, I. E. & Hinkle, R. T. *Mechanical Vibrations* (Allyn and Bacon, Boston, MA, 1963).

[26] Liu Z (2000). Locally resonant sonic materials. Science.

[27] Liu XN, Hu GK, Huang GL, Sun CT (2011). An elastic metamaterial with simultaneously negative mass density and bulk modulus. Appl. Phys. Lett..

[28] Yan X, Zhu R, Huang G, Yuan FG (2013). Focusing guided waves using surface bonded elastic metamaterials. Appl. Phys. Lett..

[29] Oudich M, Djafari-Rouhani B, Pennec Y, Assouar MB, Bonello B (2014). Negative effective mass density of acoustic metamaterial plate decorated with low frequency resonant pillars. J. Appl. Phys..

[30] Lee H, Oh JH, Seung HM, Cho SH, Kim YY (2016). Extreme stiffness hyperbolic elastic metamaterial for total transmission subwavelength imaging. Sci. Rep..

[31] Yeum CM, Sohn H, Ihn JB (2011). Lamb wave mode decomposition using concentric ring and circular piezoelectric transducers. Wave Motion.

[32] Kim HJ, Lee JS, Kim HW, Lee HS, Kim YY (2014). Numerical simulation of guided waves using equivalent source model of magnetostrictive patch transducers. Smart Mater. Struct..

[33] Vives, A. A. *Piezoelectric Transducers and Applications* (Springer Science & Business Media, Berlin, 2008).

[34] Kim K, Park CI, Lee H, Kim YY (2018). Datasets of near-zero effective impedance with finite phase velocity for sensing and actuation enhancement by resonator pairing. Figshare.

