# Supplementary material for: Near-zero effective impedance with finite phase velocity for sensing and actuation enhancement by resonator pairing
Source: Nat Commun. 2018 Dec 10;9:5255. doi: 10.1038/s41467-018-07697-7 (PMC6288096; doi:10.1038/s41467-018-07697-7)
Supplement: Supplementary file 1 — Supplementary Information [file 41467_2018_7697_MOESM1_ESM.pdf]

Supplementary Information: Near-Zero Effective Impedance with Finite Phase Velocity for Sensing and Actuation Enhancement by Resonator Pairing

Kim, K.[1] ***et* al.**

[1]*School of Mechanical and Aerospace Engineering, Seoul National University, 599 Gwanak-ro, Gwanak-gu, Seoul 151-744, Korea*

Supplementary Note 1: Detailed analysis for the equivalent system

This section is devoted to the derivation of the effective parameters z and W', and the functions $g(\omega)$ and $h(\omega)$. For this derivation, it is convenient to write $g(\omega)$ and $h(\omega)$ as

$$\frac{\tilde{u}_1}{u_1} = \frac{\tilde{u}_2}{u_2} = \frac{\tilde{u}_3}{u_3} \equiv g(\omega) = |g(\omega)|\,\mathrm{e}^{\mathrm{i}\theta}, \tag{1}$$

$$\frac{\tilde{F}_{\mathrm{inp}}}{F_{\mathrm{inp}}} = h(\omega) = |h(\omega)|\,\mathrm{e}^{\mathrm{i}\gamma}. \tag{2}$$

When equations (1–2) are substituted into equation (11) in the main manuscript, it reduces to equation (2) in the main manuscript. Likewise, equation (12) in the main manuscript can be written in terms of u_1, u_2, and F_{inp} as

$$-\mathrm{i}z\omega \frac{g(\omega)}{h(\omega)}\left(u_1\mathrm{e}^{-\mathrm{i}kL} + u_1\mathrm{e}^{\mathrm{i}kL}\right) = F_{\mathrm{inp}} - \mathrm{i}z\omega \frac{g(\omega)}{h(\omega)}\left(u_2\mathrm{e}^{-\mathrm{i}kL} - u_3\mathrm{e}^{\mathrm{i}kL}\right). \tag{3}$$

Equation (3) can be further reduced to equation (3) in the main manuscript if the following relation holds:

$$\frac{g(\omega)}{h(\omega)} = \frac{z_0}{z}. \tag{4}$$

Because the input power flow should be the same for the two systems in consideration, the following relation must hold:

$$P_{\mathrm{in}}\big|_{\mathrm{original}} = P_{\mathrm{in}}\big|_{\mathrm{equiv}}, \tag{5}$$

where

$$\begin{aligned} P_{\mathrm{in}}\big|_{\mathrm{original}} &= \frac{1}{2}\mathrm{Re}\left\{F_{\mathrm{original}} v^*_{\mathrm{original}}\right\}_{x=L} = \frac{1}{2}\mathrm{Re}\left\{F_{\mathrm{inp}}\left[\mathrm{i}\omega(u_1\mathrm{e}^{-\mathrm{i}kL} - u_1\mathrm{e}^{\mathrm{i}kL})\right]^*\right\} \\ &= \frac{1}{2}\mathrm{Re}\left\{F_{\mathrm{inp}} 2\omega \sin kL\, |u_1|\, \mathrm{e}^{-\mathrm{i}\eta}\right\} = F_{\mathrm{inp}}\omega \sin kL\, |u_1| \cos\eta \end{aligned}, \tag{6}$$

and

$$\begin{aligned} P_{\mathrm{in}}\big|_{\mathrm{equiv}} = P_{\mathrm{in}}\big|_{\mathrm{equiv}} &= \frac{1}{2}\mathrm{Re}\left\{F_{\mathrm{equiv}} v^*_{\mathrm{equiv}}\right\}_{x=L} \\ &= \frac{1}{2}\mathrm{Re}\left\{F_{\mathrm{inp}} h(\omega)\left[\mathrm{i}\omega g(\omega)(u_1\mathrm{e}^{-\mathrm{i}kL} - u_1\mathrm{e}^{\mathrm{i}kL})\right]^*\right\}. \\ &= F_{\mathrm{inp}}\omega \sin kL\, |u_1| \cos(\eta + \theta - \gamma)\, |h(\omega)|\, |g(\omega)| \end{aligned} \tag{7}$$

In equations (5–7), v denotes the particle velocity while $F_{\mathrm{original}} = F_{\mathrm{inp}}\mathrm{e}^{\mathrm{i}\omega t}$ and $F_{\mathrm{equiv}} = \tilde{F}_{\mathrm{inp}}\mathrm{e}^{\mathrm{i}\omega t}$. The symbol * denotes the complex conjugate and $u_1 = |u_1|\,\mathrm{e}^{\mathrm{i}\eta}$.

From equations (5–7), one can find

$$|h(\omega)|\,|g(\omega)| = 1, \tag{8}$$

$$\theta = \gamma. \tag{9}$$

By using equations (4, 8, 9), $g(\omega)$ and $h(\omega)$ can be found as

$$g(\omega) = \sqrt{\frac{z_0}{z}} e^{i\theta}, \tag{10}$$

$$h(\omega) = \sqrt{\frac{z}{z_0}} e^{i\theta}, \tag{11}$$

where z and θ are yet to be determined.

To determine z and θ, we eliminate $\tilde{U}$ in equations (13, 14) in the main manuscript and obtain

$$\frac{\tilde{u}_3 e^{ikW'}}{\tilde{u}_2 e^{-ikW'}} = \frac{z - z_0}{z + z_0}. \tag{12}$$

Using equation (16) in the main manuscript, equation (12) can be reduced to

$$\frac{u_3 e^{ikW'}}{u_2 e^{-ikW'}} = \frac{z - z_0}{z + z_0}. \tag{13}$$

From equation (6) in the main manuscript, it is possible to derive the following relation:

$$s(u_R - u_Q) = \frac{ms\omega^2}{s - m\omega^2} u_Q = \frac{s\omega^2}{\omega_R^2 - \omega^2} u_Q. \tag{14}$$

Combining equations (4), (5) in the main manuscript, and (14) yields

$$\begin{aligned} -iz_0\omega\left(u_2 e^{-ikW} - u_3 e^{ikW}\right) &= \left(\frac{s\omega^2}{\omega_R^2 - \omega^2} - iz_0\omega\right) u_Q \\ &= \left(\frac{s\omega^2}{\omega_R^2 - \omega^2} - iz_0\omega\right)\left(u_2 e^{-ikW} + u_3 e^{ikW}\right) \end{aligned}. \tag{15}$$

By rearranging equation (15), the following result can be obtained:

$$\frac{u_3 e^{ikW}}{u_2 e^{-ikW}} = \frac{s\omega}{2iz_0(\omega_R^2 - \omega^2) - s\omega} \equiv r = |r| e^{i\beta}. \tag{16}$$

From equations (13) and (16),

$$\frac{z}{z_0} = \frac{1 + re^{2ik(W'-W)}}{1 - re^{2ik(W'-W)}} = \frac{1 + |r| e^{i[\beta + 2k(W'-W)]}}{1 - |r| e^{i[\beta + 2k(W'-W)]}}. \tag{17}$$

To show that z is real-valued, we use the fact that the power conservation should be valid through $x = W$ for the original system and through $x = W'$ for the equivalent systems:

$$z_0 |u_2|^2 = z_0 |u_3|^2 + z_0 |U|^2 \quad \text{for the original system,} \tag{18}$$

$$\mathrm{Re}(z) |\tilde{u}_2|^2 = \mathrm{Re}(z) |\tilde{u}_3|^2 + z_0 |\tilde{U}|^2 \quad \text{for the equivalent system.} \tag{19}$$

Substituting equations (15–16) in the main manuscript and equation (10) into (19) yields

$$\frac{\mathrm{Re}(z)}{|z|} |u_2|^2 = \frac{\mathrm{Re}(z)}{|z|} |u_3|^2 + |U|^2. \tag{20}$$

Comparing equations (18) and (20) suggests that z should be positive real-valued. Therefore, z can be positive real when $\beta + 2k(W' - W) = 2n\pi$ or $(2n+1)\pi$, where n is an integer.

Now we consider first the case when $\beta + 2k(W' - W) = 2n\pi$ (n: integer), i.e.,

$$W' = W + \frac{1}{2k}\left(2n\pi - \beta\right). \tag{21}$$

In this case, z is given by

$$\frac{z}{z_0} = \frac{1+|r|}{1-|r|} > 1. \tag{22}$$

When $\beta + 2k(W' - W) = (2n+1)\pi$, i.e.,

$$W' = W + \frac{1}{2k}\left[(2n+1)\pi - \beta\right], \tag{23}$$

z is given by

$$\frac{z}{z_0} = \frac{1-|r|}{1+|r|} < 1. \tag{24}$$

In our study, the result in equation (24) will be used because we are interested in $z < z_0$.

In the remaining part, we will derive equations (20a, b) in the main manuscript. For this derivation, we utilize the following relation, which can be obtained from equations (13–16) in the main manuscript and equation (24):

$$\frac{\tilde{U}}{\tilde{u}_2} = \frac{U}{gu_2} = \frac{2z}{z_0 + z} = 1 - |r|. \tag{25}$$

Using equation (4) in the main manuscript and equation (16), it is possible to obtain

$$\frac{U}{u_2} = 1 + \frac{u_3}{u_2}\mathrm{e}^{2ikW} = 1 + r. \tag{26}$$

Then, $g(\omega)$ can be explicitly written only in terms of r as

$$g(\omega) = \frac{1+r}{1-|r|} = \frac{1+|r|(\cos\beta + \mathrm{i}\sin\beta)}{1-|r|} \equiv |g(\omega)|\mathrm{e}^{\mathrm{i}\theta}. \tag{27}$$

where r is expressed as $r = |r|\cos\beta + \mathrm{i}|r|\sin\beta$. If equation (16) and equation (26) are substituted into equation (18), the following relation can be derived:

$$1 = |r|^2 + |1+r|^2. \tag{28}$$

The substitution of $r = |r|\cos\beta + \mathrm{i}|r|\sin\beta$ into equation (28) yields an expression from which β can be determined:

$$\cos\beta = -|r|. \tag{29}$$

Taking the absolute value of both sides of equation (27) and using equation (28), $|g(\omega)|$ is found to be

$$|g(\omega)| = \frac{|1+r|}{1-|r|} = \frac{\sqrt{1-|r|^2}}{1-|r|} = \sqrt{\frac{1+|r|}{1-|r|}} = \sqrt{\frac{z_0}{z}}. \tag{30}$$

The phase θ of $g(\omega)$ can be found from equations (27) and (29) as

$$\tan\theta = \frac{|r|\sin\beta}{1+|r|\cos\beta} = \frac{-\cos\beta\sin\beta}{1-\cos^2\beta} = -\frac{1}{\tan\beta},$$

$$\tan\theta = -\frac{1}{\tan\beta}. \quad (31)$$

Referring to the definition of r given in equation (16), the range of β varies depending on ω as

$$\begin{aligned} &\pi < \beta < 3\pi/2 \quad \text{when} \quad \omega < \omega_R, \\ &\pi/2 < \beta < \pi \quad \text{when} \quad \omega > \omega_R. \end{aligned} \quad (32)$$

Therefore, θ is determined as

$$\theta = \begin{cases} \beta - \pi/2, & (\omega < \omega_R) \\ \beta + \pi/2, & (\omega > \omega_R) \end{cases}. \quad (33)$$

More compactly, $\theta = \beta + p\pi/2$ with $p = \text{sign}(\omega - \omega_R)$, as given in equation (20) in the main manuscript.

Supplementary Note 2: The finite element analysis to extract mass and stiffness of the resonator

To estimate the mass m and stiffness s of the C-shaped resonator, its eigenfrequency f_R and displacement u_R under a static force are used. As mentioned in the manuscript, the lowest eigenfrequency of the C-shaped resonator was found to be 93.1 kHz for the geometry shown in Fig. 4 (t_R = 3 mm, w_R = 6 mm, b_R = 1.5 mm, and h_R = 4.5 mm) and the corresponding eigenmode is shown in Supplementary Figure 1(a). The eigenmode confirms that the dominant motion of the resonator at the resonance frequency is along the x direction. Furthermore, the analysis of the eigenmode shape suggests that the pillar part of the resonator works as a spring, while its upper body part works as a mass in a mass-spring system. Therefore, it is possible to apply $f_x/2$ to each of the mass parts of the resonator for the evaluation of s, as illustrated in Supplementary Figure 1(b). Note that the application of force $f_x/2$ to each mass parts is equivalent to the application of f_x to the entire resonator. Here, the applied force is prescribed as a uniformly distributed force everywhere in the mass body part. The average horizontal displacement of the mass part u_R under the force is obtained by the detailed finite element analysis. By this procedure, the stiffness value of s is estimated to be 35.3 GN/m and the mass m is then calculated from $m = s/(2\pi f_R)^2$ = 103.0 g.

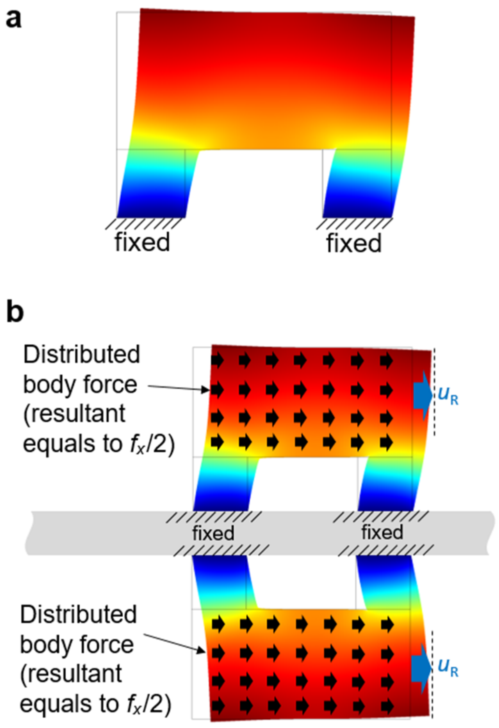

Supplementary Figure 1. **Finite element analysis for the parameter estimation of the C-shaped resonator.** (a) Frontal view of the lowest eigenmode of the resonator and (b) deformed shape of the pillars of C-shaped resonator under an applied force (f_x) along the x axis.
